# Stent Thrombosis Patients with Hyporesponsiveness to Clopidogrel, Prasugrel, and Ticagrelor: A Case Series Using Short Thromboelastography

**DOI:** 10.1155/2016/2096181

**Published:** 2016-10-09

**Authors:** Bartosz Olechowski, Alexander Ashby, Nalyaka Sambu, Michael Mahmoudi, Nick Curzen

**Affiliations:** Wessex Cardiothoracic Centre, University Hospital Southampton NHS Foundation Trust, Southampton, UK

## Abstract

Patients after percutaneous coronary intervention (PCI) with stent implantation and functional hyporesponsiveness to P_2_Y_12_ inhibitors are at higher risk of ischaemic events, particularly stent thrombosis (ST). It is currently not routine practice to assess the functional response to these agents. However, concern over functional hyporesponsiveness to clopidogrel has led to widespread uptake of prasugrel and ticagrelor as the default P_2_Y_12_ inhibitor after stent implantation in patients with acute coronary syndrome. Here we report, for the first time, 3 cases in which patients who have had ST exhibit hyporesponsiveness to clopidogrel, prasugrel, and ticagrelor.

## 1. Introduction

Stent thrombosis (ST) is a major complication of percutaneous coronary intervention (PCI), occurring in 2.0–2.9% of patients within 22 months [1]. Although uncommon, ST is associated with significant mortality of up to 45% [2]. Dual antiplatelet therapy (APT) with aspirin and P_2_Y_12_ inhibitor has become the default strategy in patients undergoing coronary stent implantation to reduce the risk of ST. However, a cohort of patients may have an inadequate functional response to P_2_Y_12_ [3, 4] and are more likely to sustain ischaemic events including ST [5].

There is particular concern about clopidogrel in this regard [6, 7]. The established link between functional hyporesponsiveness to clopidogrel and ischaemic events, including ST, in patients receiving coronary stents has triggered the development of more potent and faster-acting P_2_Y_12_ inhibitors. Two large randomised trials have demonstrated reduction in ischaemic endpoints for prasugrel and ticagrelor when compared to clopidogrel in acute coronary syndrome (ACS) patients undergoing PCI, albeit at the price of increased bleeding [8, 9]. In response to these data and earlier studies demonstrating quicker onset and more potent and more homogeneous responses of healthy volunteers and stable patients to prasugrel and ticagrelor compared to clopidogrel, many PCI centres in the UK have switched from clopidogrel to either prasugrel or ticagrelor as their default. Interestingly the incidence of prasugrel hyporesponsiveness is estimated to be 25% using flow cytometric analysis of intraplatelet vasodilator-stimulated phosphoprotein (VASP) phosphorylation in ACS patients [10, 11]. In the CREST registry, out of 6 patients who were found to be hyporesponsive to prasugrel, only 3 responded adequately to ticagrelor [6].

We present for the first time 3 cases who had experienced definite ST after drug eluting stent (DES) implantation who demonstrated functional hyporesponsiveness to clopidogrel, prasugrel, and ticagrelor, using a previously well validated test, short thromboelastography (sTEG) [12–15]. sTEG uses a novel parameter, percentage clotting inhibition (%CI) in the AA or ADP channel for clotting inhibition by aspirin or P_2_Y_12_ inhibitors, respectively. The formula for %CI by aspirin is 100 − (AUC15(AA)/AUC15(Thrombin) × 100) and for %CI by P_2_Y_12_ inhibitors is 100 − (AUC15(ADP)/AUC15(Thrombin) × 100) [14]. Threshold %CI of <50 in the AA channel and <30 in the ADP channel was used to define hyporesponsiveness to aspirin and P_2_Y_12_ inhibitors, respectively.

## 2. Case Report

**Figure 1:**
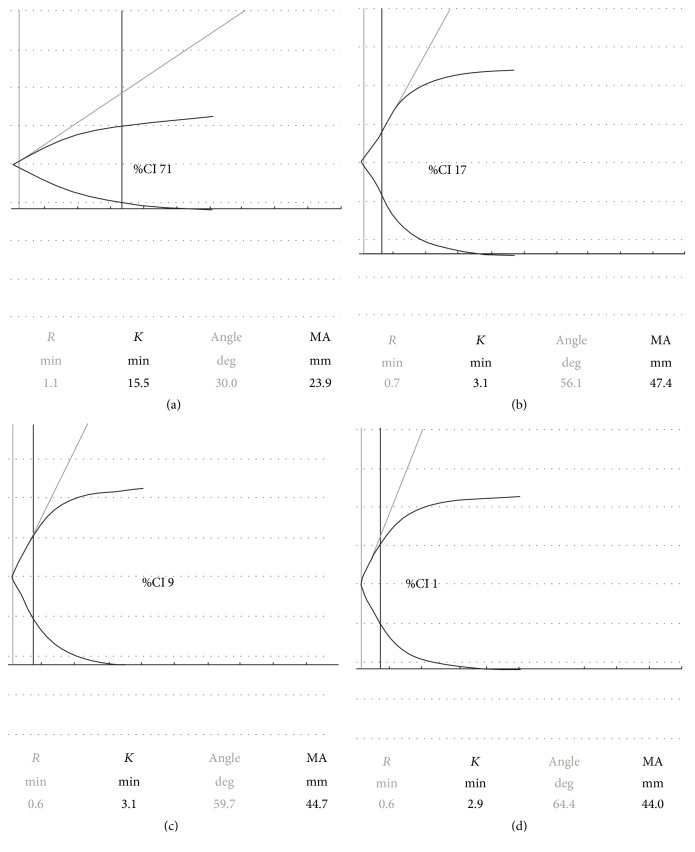
Short thromboelastography traces showing adequate response to aspirin 150 mg daily and hyporesponse to P_2_Y_12_ inhibitors in first patient. (a) Patient 1 clotting response to AA when on 150 mg daily aspirin, producing a %CI(AA) of 71, an adequate response to aspirin. (b) Patient 1 clotting response to ADP when on 75 mg clopidogrel daily, producing a %CI(ADP) of 17 (nonresponse to clopidogrel). (c) Patient 1 clotting response to ADP when on 10 mg prasugrel daily, producing a %CI(ADP) of 9 (nonresponse to prasugrel). (d) Patient 1 clotting response to ADP when on 90 mg ticagrelor twice daily, producing a %CI(ADP) of 1 (nonresponse to ticagrelor).

Patient 1 is a 74-year-old male with type 2 diabetes mellitus and previous anterior ST elevation myocardial infarction (STEMI) treated with a single drug eluting stent (DES) in the circumflex artery. He presented with proximal stent occlusion 2043 days after his index PCI while on aspirin 75 mg once daily. He was successfully treated with plain old balloon angioplasty (POBA) and bare metal stent (BMS) insertion. Subsequently he underwent platelet function testing using sTEG. Initially our patient was started on aspirin 150 mg daily and clopidogrel 75 mg daily. Forty-two days later, the assay revealed an adequate response to aspirin (%CI 71) but suboptimal response to clopidogrel (%CI 17). Therefore, prasugrel 5 mg daily was commenced as patient was borderline for age group with no initial loading. Once more the reading showed inadequate response to prasugrel 5 mg daily (%CI −7) after 63 days of treatment and the dose was uptitrated to 10 mg daily. Subsequent test, 105 days later, revealed suboptimal response again (%CI 9). As a result, the patient was commenced on ticagrelor 90 mg twice daily without initial loading and retested after 85 days of treatment. Similarly, his reading revealed hyporesponse (%CI 1) (Figure 1). Due to development of dyspnoea while on ticagrelor, the patient was finally left on prasugrel 10 mg daily for life. After this episode, he was treated with cardiac resynchronisation therapy and defibrillation due to severe ischaemic cardiomyopathy but is currently alive, having suffered no further ST or other ischaemic events.

**Figure 2:**
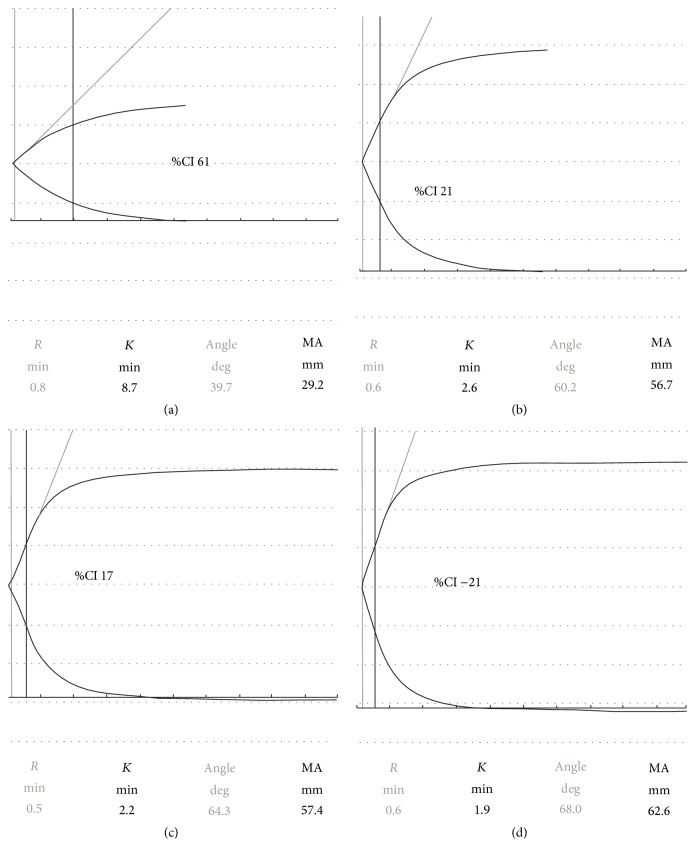
Short thromboelastography traces showing adequate response to aspirin 75 mg daily and hyporesponse to P_2_Y_12_ inhibitors in second patient. (a) Patient 2 clotting response to AA when on 75 mg daily aspirin, producing a %CI(AA) of 61, an adequate response to aspirin. (b) Patient 2 clotting response to ADP when on 75 mg clopidogrel daily, producing a %CI(ADP) of 21 (nonresponse to clopidogrel). (c) Patient 2 clotting response to ADP when on 10 mg prasugrel daily, producing a %CI(ADP) of 17 (nonresponse to prasugrel). (d) Patient 2 clotting response to ADP when on 90 mg ticagrelor twice daily, producing a %CI(ADP) of −21 (nonresponse to ticagrelor).

Patient 2 is a 62-year-old male smoker with hyperlipidaemia and positive family history for premature coronary artery disease who originally presented with a non-ST-elevation myocardial infarction (NSTEMI) for which he had three DES implanted in the left anterior descending artery (LAD). He represented with anterior STEMI due to ST 795 days after his index admission while on aspirin 75 mg daily. He was treated with intravascular ultrasound guided POBA. Platelet function testing using sTEG demonstrated an inadequate response to P_2_Y_12_ receptor inhibitors. Initial response to aspirin 75 mg daily appeared adequate (%CI 61); however response to clopidogrel 75 mg daily was suboptimal (%CI 21) after 36 days of treatment. As a result, the patient was commenced on prasugrel 10 mg daily with no initial loading. Testing, after 70 days of treatment, again revealed hyporesponsiveness (%CI 17). Finally, ticagrelor 90 mg bd was introduced, with no initial loading, with similar effect (%CI −21) after 263 days of treatment (Figure 2). Ultimately, he was maintained on ticagrelor 90 mg twice daily for life and has suffered no further ST or ischaemic events to date.

Patient 3 is a 59-year-old male smoker with hypercholesterolaemia and a positive family history for premature coronary artery disease, who initially had PCI with two DES to the right coronary artery (RCA) and LAD in the context of a NSTEMI. He represented 671 days later with ST while on aspirin 150 mg daily. Both vessels required treatment due to acute thrombotic occlusion and four further DES were implanted. He was proven to have adequate response to aspirin 150 mg daily (%CI 82) but suboptimal response to thienopyridines. Clopidogrel 75 mg with inadequate response (%CI 8) after 19 days of treatment was replaced by prasugrel 10 mg daily with no initial loading, with again suboptimal response (%CI 9) after 56 days of usage. Finally, ticagrelor 90 mg twice daily was commenced without initial loading but similarly was proven to have inadequate response (%CI 24) after 21 days of treatment. As response to ticagrelor was better than to prasugrel, the decision was made for him to remain on ticagrelor for 12 months. Patient 3 has suffered no further ischaemic events to our knowledge.

**Figure 3:**
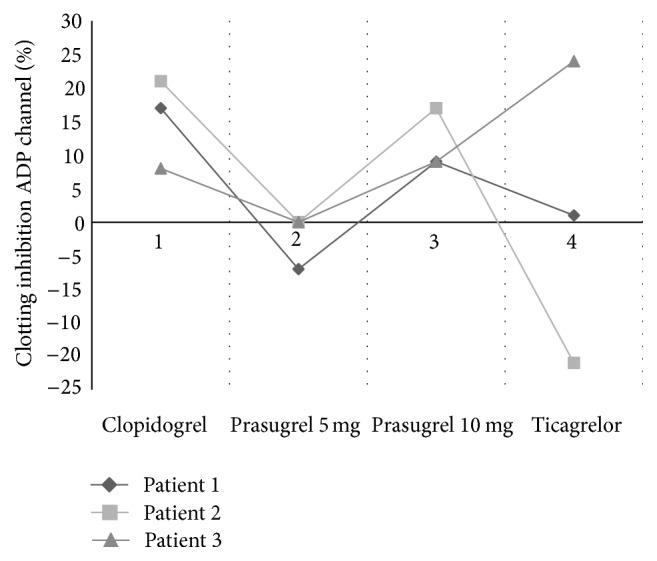
Variability in % clotting inhibition following medication adjustments in patients found to be hyporesponsive at initial and successive short TEG tests following stent thrombosis. Horizontal dotted line signifies a sufficient response.

The %CIn(ADP) for each of our patients are demonstrated as in Figure 3.

## 3. Discussion

Our case series demonstrates three cases of patients with acute stent thrombosis who were found to be hyporesponsive to all three commonly available P_2_Y_12_ receptor inhibitors. To the best of our knowledge, this is the first time that a series of such cases has been described. Two previously published case reports have illustrated patients with dual thienopyridine resistance to clopidogrel and prasugrel [16, 17]. In both cases, the patient was subsequently shown to have an adequate response to the ticagrelor. Orban et al. suggested that the response to ticagrelor may be due to its properties as an active drug, compared with prasugrel and clopidogrel which are prodrugs requiring hepatic cytochrome bioactivation prior to P_2_Y_12_ inhibition [17].

Based upon both early studies reporting superior potency, speed of onset and consistency of responses to prasugrel and ticagrelor versus clopidogrel in both volunteers and stable patients [18, 19], and subsequent large scale randomised trials, in many PCI centres, prasugrel and ticagrelor have become the default P_2_Y_12_ agent. The assumption from some interventionalists is that prasugrel and ticagrelor are not associated with functional hyporesponsiveness (also known as “resistance”). However, recent data suggest that functional resistance does indeed occur in association with these agents [11, 20].

Our case series describe for the first time the concept of functional resistance to all three commonly available P_2_Y_12_ inhibitors. These data were obtained using short TEG, a well described and validated modification of TEG platelet mapping assay [10–13]. Our group has previously described the reproducibility of sTEG and has demonstrated the value of comparing the ADP-induced clotting response to that achieved using kaolin stimulation. This method has the advantage that there is a built-in reference, both numerical and visual, of the strength of the clot in response to ADP compared to the maximum clot strength achieved by kaolin. We have also used sTEG to describe discrepancies between these results from Verify Now assay [21, 22].

One limitation of our case series is that we were not able to objectively prove patients' compliance with medications, although we appreciate that it might have implications on final management strategy. In addition to this, no other platelet function assays (i.e., Verify Now) were used to confirm our results.

These cases suggest that some patients experiencing ST may exhibit functional resistance to all 3 of the currently available oral P_2_Y_12_ inhibitors. As well as adding weight to the argument in favour of measuring individual responses to P_2_Y_12_ inhibitors, with the intention of possibly avoiding stents in patients who do not respond to them, it also raises interesting questions about the mechanism of hyporesponsiveness.

Further data are required to investigate whether this observation in such patients could be due to a paucity of receptor numbers, a functional flaw in the receptor activation, or perhaps even a lack of availability of such drugs at the receptor.
